# *Staphylococcus aureus* Alpha-Toxin in Deep Tracheal Aspirates—Preliminary Evidence for Its Presence in the Lungs of Sepsis Patients

**DOI:** 10.3390/toxins14070450

**Published:** 2022-06-30

**Authors:** Sabine Ziesemer, Sven-Olaf Kuhn, Anke Hahnenkamp, Manuela Gerber, Elvira Lutjanov, Matthias Gruendling, Jan-Peter Hildebrandt

**Affiliations:** 1 Animal Physiology and Biochemistry, University of Greifswald, Felix Hausdorff-Strasse 1, D-17489 Greifswald, Germany; sabine.ziesemer@uni-greifswald.de (S.Z.); elvira.lutjanov@uni-greifswald.de (E.L.); 2 Department of Anesthesiology, University Hospital Greifswald, Ferdinand Sauerbruch-Strasse, D-17475 Greifswald, Germany; sven-olaf.kuhn@med.uni-greifswald.de (S.-O.K.); anke.hahnenkamp@med.uni-greifswald.de (A.H.); manuela.gerber@med.uni-greifswald.de (M.G.); matthias.gruendling@med.uni-greifswald.de (M.G.)

**Keywords:** *Staphylococcus aureus*, alpha-toxin, deep tracheal aspirate, sepsis patients

## Abstract

The pore forming alpha-toxin (hemolysin A, Hla) of *Staphylococcus aureus* (*S. aureus*) is a major virulence factor with relevance for the pathogenicity of this bacterium, which is involved in many cases of pneumonia and sepsis in humans. Until now, the presence of Hla in the body fluids of potentially infected humans could only be shown indirectly, e.g., by the presence of antibodies against Hla in serum samples or by hemolysis testing on blood agar plates of bacterial culture supernatants of the clinical isolates. In addition, nothing was known about the concentrations of Hla actually reached in the body fluids of the infected hosts. Western blot analyses on 36 samples of deep tracheal aspirates (DTA) isolated from 22 hospitalized sepsis patients using primary antibodies against different epitopes of the Hla molecule resulted in the identification of six samples from five patients containing monomeric Hla (approx. 33 kDa). Two of these samples showed also signals at the molecular mass of heptameric Hla (232 kDa). Semiquantitative analyses of the samples revealed that the concentrations of monomeric Hla ranged from 16 to 3200 ng/mL. This is, to our knowledge, the first study directly showing the presence of *S. aureus* Hla in samples of airway surface liquid in human patients.

## 1. Introduction

*Staphylococcus aureus* (*S. aureus*) is a commensal bacterium in humans. *S. aureus* colonizes the skin as well as the anterior nares. Approximately 20% of individuals are persistent nasal carriers, 30% are intermittent carriers, and 50% are non-carriers [1]. Occasionally, this bacterium may cause clinically important conditions such as skin infections, endocarditis, osteomyelitis, sinusitis, pneumonia, or sepsis [1]. Because of the increasing resistance to antibiotics (e.g., methicillin-resistant *S. aureus*, MRSA) treatment of these ailments has become a major problem [2,3].

The pathogenicity of *S. aureus* depends on the ability of the bacteria to express different virulence factors. One major virulence factor that strongly determines the morbidity as well as the mortality of *S. aureus*-infected organisms is the alpha-toxin (hemolysin A, Hla) [4,5]. Hla is secreted by bacteria upon reaching critical densities as a water-soluble monomer of 33.2 kDa. Upon binding to the plasma membrane of the host cell, monomers oligomerize to form a heptameric pre-pore and subsequently a 232.4 kDa transmembrane pore [6] permeable for ions and small molecules [7,8]. In airway epithelial cells, formation of such transmembrane pores results in alterations in membrane potential and cytosolic ion concentrations, modifications in cell signaling, rearrangements of the actin cytoskeleton, loss of cell–cell and cell–matrix contacts, and, ultimately, in the formation of paracellular gaps in the epithelial cell layer [9,10,11,12,13,14]. In vivo, such effects of pore formation would disrupt the barrier function of the respiratory epithelium [15], which may enable external bacteria to enter the interior of the body [7].

Detection of anti-alpha-toxin antibodies in serum samples of *S. aureus*-infected patients indicate that Hla plays an important role in pathogenicity [16]. Additionally, researchers were able to isolate bacteria from the skin, blood, or respiratory tract of infected patients that carried the Hla gene and were able to find Hla protein in the culture supernatants of these bacteria in the lab [17,18]. However, there is still no direct evidence for the presence of physiologically relevant concentrations of Hla in intact human airways colonized with *S. aureus*. The objective of the present study was to examine the presence and the levels of HIa in deep tracheal aspirates (DTA) of adult sepsis patients.

## 2. Results

**Figure 1:**
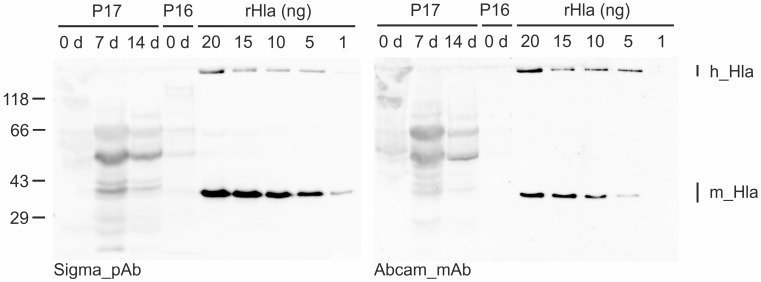
Detection and semi-quantification of Hla in DTA samples from patients (P) with sepsis by semiquantitative Western blotting. Shown is an example blot that was incubated with the anti-Hla polyclonal antibody from Sigma (image on the left) and, upon stripping, with the anti-Hla monoclonal antibody from Abcam (image on the right). Five lanes of this blot were loaded with different amounts (1 to 20 ng) of recombinant Hla (rHla) to compare their signal intensities with those of the DTA samples for semi-quantification of the Hla content of each sample. Note that the Sigma antibody is slightly more sensitive compared with the Abcam antibody (different signal intensities in the rHla lanes). m_Hla—monomeric Hla; h_Hla—heptameric Hla.

In total, 36 DTA samples taken from 22 septic patients were analyzed (Table S1). Semiquantitative Western blot analyses of these samples showed the presence of Hla in six samples from five patients (monomeric: 33 kDa; heptameric: 232 kDa) (Figure 1; Table 1). All other samples were free of Hla or may have contained amounts of Hla that were below the detection limit of the Western blot system (approx. 0.1 ng in a 12.5 μL DTA aliquot). Additional bands that occasionally occur in the mass range of 43 to 66 kDa are most likely due to nonspecific binding of the antibodies.

Hla was detected in DTA samples from patients 5, 16, and 21 at the day of admission. Patients 1 and 17 were sampled at day 7 after hospitalization. The latter patient was probed again at day 14 after admission to the hospital, and the sample was again Hla-positive. In cases where sufficient sample volumes were available, Western blotting was repeated using dilution series of recombinant Hla in different ranges to make sure that semi-quantification was adequate. In such cases we also changed the sequence of the primary antibodies to exclude technical errors (Figure S1).

Trials to estimate the amounts of monomeric Hla present in DTA samples were partially successful depending on the total sample volumes that were available. The sample volume obtained from patient 5 was so small (only a few microliters) that we were able to analyze it only once in a qualitative manner with both antibodies but could not properly quantify the Hla amounts. In the other Hla-positive samples, we found monomeric Hla concentrations of 16 (Patient 16) to 3200 ng/mL (Patient 1).

**Figure 2:**
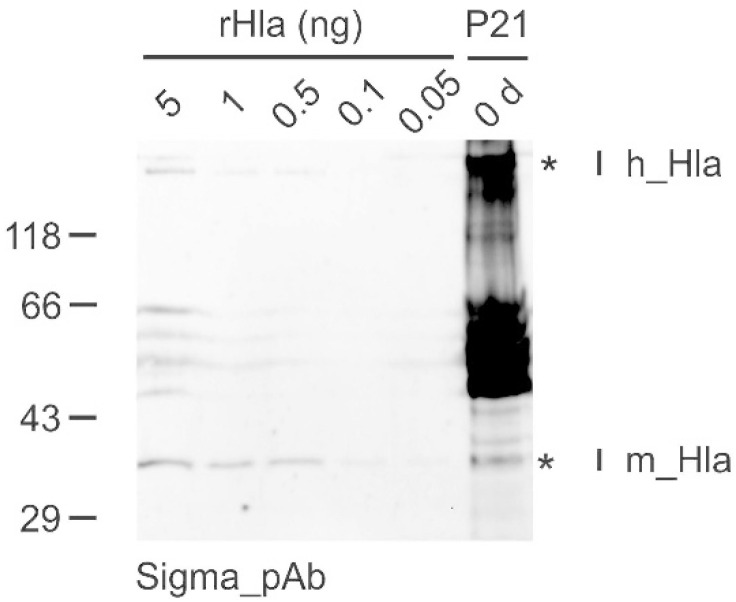
Detection of monomeric (m_Hla) as well as heptameric Hla (h_Hla) in DTA samples from patients with sepsis by Western blotting. Shown is an example blot that was incubated with the anti-Hla polyclonal antibody from Sigma. Five lanes of this blot were loaded with different amounts (0.05 to 5 ng) of rHla to compare their signal intensities with those of the DTA sample of Patient 21 (P21) for semi-quantification of the monomeric Hla content of each sample. The presence of a band at approximately 230 kDa indicates that the sample of Patient 21 contains heptameric Hla in addition to the monomeric form of the toxin. No attempt was made to quantify the heptameric Hla as this depends on the amount of cellular material in the sample. *—Bands of monomeric and multimeric Hla in the lane containing the sample obtained from Patient 21.

In two of the Hla-positive samples (Patient 1 and Patient 21), we were able to visualize Western blot bands in the high molecular mass range of approximately 230 kDa, which appeared in the same range as heptamers of recombinant Hla (Figure 2). The occurrence of such bands correlated well with the observation of the presence of tissue fragments in the samples, which were absent in the other Hla-positive samples.

## 3. Discussion

Previous studies gave indirect proof of the occurrence of Hla in humans and animals infected with Hla-producing strains of *S. aureus*, e.g., by showing the presence of Hla-reactive antibodies in serum samples [19,20,21]. However, direct proof of Hla generation during acute infections is, to our knowledge, not yet existing. This lack of evidence may have several reasons. Especially in airway infections involving *S. aureus*, high densities of these bacteria may occur only transiently, while other bacterial strains are more abundant during later phases [22,23]. Donors of these kinds of samples have usually had antibiotic treatment, which may, at least for some time, erase the bacteria but leave residual toxins detectable in some of the samples. This may explain why *S. aureus* could be cultured from only 11.3% of all sputum samples from cystic fibrosis patients [24] and may also limit the occurrence of detectable amounts of Hla in such samples. Furthermore, most of the monomeric Hla produced by *S. aureus* in the airway surface liquid (ASL) is probably readily bound to cell membranes of cells in the airways and undergoes rapid heptamerization [25]. This is likely to limit the rate of accumulation of monomeric Hla in samples of sputum, bronchoalveolar lavage fluid, or deep tracheal aspirates.

Nevertheless, we were able to directly detect Hla by semiquantitative Western blotting using antibodies against two different epitopes of *S. aureus* Hla in six samples of DTA (Table 1) from a total of 36 samples obtained from 22 sepsis patients (Table S1). Four of these samples were taken from three patients (# 16, 17, and 21) who had tested positive for *S. aureus* bacteria. In six other patients who tested positive for *S. aureus* (# 6, 8, 9, 11, 19 and 22), we did not detect any Hla in the DTA samples. These results illustrate the situation described above and may result from differences in the infection histories in the given patients. If the infection originates in bone (as in patients 6 and 11), it may well be that the lungs are free of bacteria and also free of Hla. This finding may also be explained by the presence of *S. aureus* strains in these patients which did not express Hla at all [17]. On the other hand, we detected Hla in DTA samples from two patients (# 1 and 5) who did not show any signs of *S. aureus* infections. These cases may be explained by recent antibiotic treatment that may have eliminated the bacteria leaving traces of the toxin in DTA behind. Our results, however, provide proof of principle that Hla may be produced by genetically able *S. aureus* and may accumulate to detectable concentrations in the airway liquids under conditions of acute or chronic airway infection.

In some of the Hla-positive DTA samples, we could determine the concentrations of monomeric Hla which ranged from 16 ng/mL (Patient 16) to 3200 ng/mL (Patient 1), which matches the range of Hla concentrations that are usually used for in vitro analyses of Hla effects in eukaryotic model cells [13]. The reasons for the large variations in Hla concentrations may be the same as discussed above or may be related to potential differences in the abundances of bacteria in the airways of the respective patients that had not been quantitatively determined.

In addition to the monomeric Hla (33 kDa), we found signals at 232 kDa in the Western blots of samples from patients 1 and 21 that could be attributed to heptameric transmembrane pores. Generally, we know from our routine storage of recombinant Hla that Hla monomers maintained at high concentrations at room temperature may spontaneously form heptamers even in the absence of cells at low rates. However, rapid multimerization of Hla monomers under physiological conditions seems to depend on the presence of cellular material, attachment of monomeric Hla to ADAM10 as a cell surface receptor [26,27], and the presence of sphingomyelin-rich plasma membrane areas facilitating the assembly of Hla heptamers [28]. It was obvious from visual inspection that samples from patients 1 and 21 contained small amounts of tissue material while the other Hla-positive samples were free of such materials. These considerations may explain why we did not identify Hla heptamers in the other Hla-positive samples. It would be interesting to perform a similar study on bronchioalveolar lavage fluids, as those may not contain any tissue.

To summarize, we were able to show the presence of Hla in samples of deep tracheal aspirates obtained from human sepsis patients. In Hla-positive samples, the concentration of monomeric Hla covered the range from 16 ng/mL to 3200 ng/mL. This is, to our knowledge, the first study showing directly the presence of *S. aureus* Hla in samples of tracheal aspirates in human sepsis patients and its concentration range.

## 4. Materials and Methods

### 4.1. Deep Tracheal Aspirates (DTA)

DTAs were obtained through sterile suction catheters using an aseptic technique. Samples from 22 patients infected with different types of microorganisms (Table S1) were taken directly at the day of admission and/or at days 7 or 14 after hospitalization. Aspirates were immediately stored at −80 °C upon collection. In addition, the types of microorganisms that were causative of sepsis were noted, and the pathogens present in the airways were determined by bacteriological means (especially by blood cultures).

### 4.2. Sample Preparation for the Blood Culture of Microorganisms

Blood (2 × 20 mL) was obtained from the peripheral veins of patients before treatment with antibiotics was started. It is unknown whether patients had received antibiotics prior to hospitalization. Blood (10 mL) was added to each of two culture flasks containing resins for antibiotic neutralization. Blood cultures (BACTEC™ Plus Aerobic/F and BACTEC™ Plus Anaerobic/F culture vials; Becton-Dickinson, Franklin Lakes, NJ, USA) were obtained in accordance with current blood culture guidelines [29] and incubated in an automated blood culture system (BD BACTEC FX; Becton-Dickinson) for 5 days at 37 °C. Positive blood cultures were Gram-stained, streaked onto Columbia sheep blood agar, chocolate agar, MacConkey lactose agar, and Schaedler agar (Becton-Dickinson) for overnight incubation at 37 °C, and species identification was then carried out using matrix-assisted laser desorption ionization time-of-flight mass spectrometry on a VITEK^®^MS device (bioMérieux, Marcy l’Étoile, France).

### 4.3. Antibodies

To detect Hla mono- as well as heptamers, two different primary antibodies against *S. aureus* Hla were used in semiquantitative Western blotting: (1) anti-alpha-hemolysin antibody (S7531) from Sigma (Steinheim, Germany). This polyclonal antibody was produced in rabbit using purified toxin from *S. aureus* as an immunogen. The antibody is specific for different epitopes within the same antigen;(2) anti-alpha-hemolysin antibody [8B7] (ab190467) from Abcam (Cambridge, UK). This monoclonal mouse anti-alpha-hemolysin antibody targets the N terminus of the mature Hla toxin. Goat anti-rabbit-IgG-HRP (sc-2004) or goat anti-mouse-IgG-HRP (sc-2005) obtained from Santa Cruz Biotechnology (Heidelberg, Germany) were used as secondary antibodies.

### 4.4. Recombinant Hla (rHla)

Expression and purification of recombinant *S. aureus* alpha-toxin (rHla) were performed as described previously [13,30].

### 4.5. Western Blot Analysis

Approximately 100 μL of each DTA sample was diluted 1:2 in SDS sample buffer (50 mmol/L Tris, 1% SDS, 0.2% bromphenolblue, 4% β-mercaptoethanol, 40% glycerol, and pH 6.8) and heated to 95 °C for 5 min. The samples were loaded on 10% SDS electrophoresis gels in a minigel apparatus (BioRad, Munich, Germany) and subjected to 1D-gelelectrophoresis, as described previously [27]. ROTI^®^Mark STANDARD T851.2, Roth, Karlsruhe, Germany) was used as a molecular weight marker to determine the apparent molecular masses of protein(s) of interest. One lane on each gel was loaded with rHla for band identification. To determine the amounts of Hla present in the samples, a range of concentrations of recombinant Hla was loaded in several lanes next to the DTA samples.

The proteins were blotted from the gels onto nitrocellulose membranes (HP40, Roth, Karlsruhe, Germany) by wet blotting, as described previously [27]. Quantification of Hla was performed by Western blotting using one of two different Hla primary antibodies (1:3333), HRP-linked secondary antibodies (1:6000), and enhanced luminescence reagents (Biozym, Oldendorf, Germany). Chemiluminescent signals were detected using an Intas Chemostar ECL imager (Intas, Goettingen, Germany). Band signal intensities were assessed by densitometry using Phoretix 1 D (Nonlinear Dynamics, Newcastle upon Tyne, UK). Upon imaging the band obtained with the first Hla antibody, the blot was stripped in a buffer solution (47.47 mmol/L Tris, 2% (*v*/*v*) SDS, 1% (*v*/*v*) β-mercaptoethanol, and pH 6.7) for 20 min at 50 °C. Stripping efficiency was verified by incubating the membrane again with chemiluminescence detection reagent and luminescence imaging. The proteins on the membrane were then re-probed with the second type of Hla antibody to ensure signal specificity.

## Figures and Tables

**Table 1:** List of patient samples in which Hla mono- or heptamers were detected, and semi-quantification of the respective Hla contents (- no Hla heptamers detected). Samples were taken from the patients at hospitalization (day 0) or several days after admission. The amounts of Hla mono- and heptamers in the samples were determined by semiquantitative Western blotting using Hla-specific antibodies and a dilution series of recombinant Hla in neighboring lanes on the same gel. The sample volume obtained from Patient 5 was too small to allow quantification of Hla (n.d.—not determined). Patient #—patient number.

Patient #	Day of Sampling	Detection of *S. aureus* in Patient	Hla Detected	Monomeric Hla (ng/mL DTA)
1	7	no	yes	3173
5	0	no	yes	n.d.
16	0	yes	yes	16
17	7	yes	yes	128
	14	yes	yes	56
21	0	yes	yes	384

## Data Availability

The data presented in this study are available in this article and Supplementary material.

## Supplementary Materials

The following supporting information can be downloaded at: https://www.mdpi.com/article/10.3390/toxins14070450/s1, Figure S1. Detection and semi-quantification of monomeric Hla in deep tracheal aspirate (DTA) samples from patients with sepsis by semi-quantitative Western blotting. Shown is an example blot that had been incubated with the anti-Hla monoclonal antibody from Abcam (image on the left) and, upon stripping, with the anti-Hla polyclonal antibody from Sigma (image on the right). Five lanes of this blot were loaded with different amounts (0.1 to 5 ng) of recombinant Hla to compare their signal intensities with those of the DTA samples for semi-quantification of Hla content of each sample. The patient numbers (P) correspond to those given in Supplemental Table S1. The stars point to positive signals: m_Hla—monomeric Hla; h_Hla—heptameric Hla; Figure S2. Detection and semi-quantification of monomeric Hla in deep tracheal aspirate (DTA) samples from patients with sepsis by semi-quantitative Western blotting. Shown are blots containing Hla-positive samples that had been incubated with the anti-Hla polyclonal antibody from Sigma. Five lanes of the blot on the left were loaded with different amounts (0.05 to 5 ng) of recombinant Hla to compare their signal intensities with those of the DTA samples for semi-quantification of Hla content of each sample. The patient numbers (P) correspond to those given in Supplemental Table S1. The stars point to bands that correspond to monomeric Hla. m_Hla—monomeric Hla; h_Hla—heptameric Hla. Table S1: List of deep tracheal aspirate (DTA) samples from hospitalized septic patients.
