# Supplementary material for: Staphylococcus aureus Alpha-Toxin in Deep Tracheal Aspirates—Preliminary Evidence for Its Presence in the Lungs of Sepsis Patients
Source: Toxins (Basel). 2022 Jun 30;14(7):450. doi: 10.3390/toxins14070450 (PMC9320683; doi:10.3390/toxins14070450)
Supplement: Supplementary file 1 [file toxins-14-00450-s001.zip › toxins-1731664-supplementary.pdf]

toxins

Supplementary Materials: *Staphylococcus aureus* Alpha-Toxin in Deep Tracheal Aspirates—Preliminary Evidence for Its Presence in the Lungs of Sepsis Patients

Sabine Ziesemer, Sven-Olaf Kuhn, Anke Hahnenkamp, Manuela Gerber, Elvira Lutjanov, Matthias Gruendling and Jan-Peter Hildebrandt

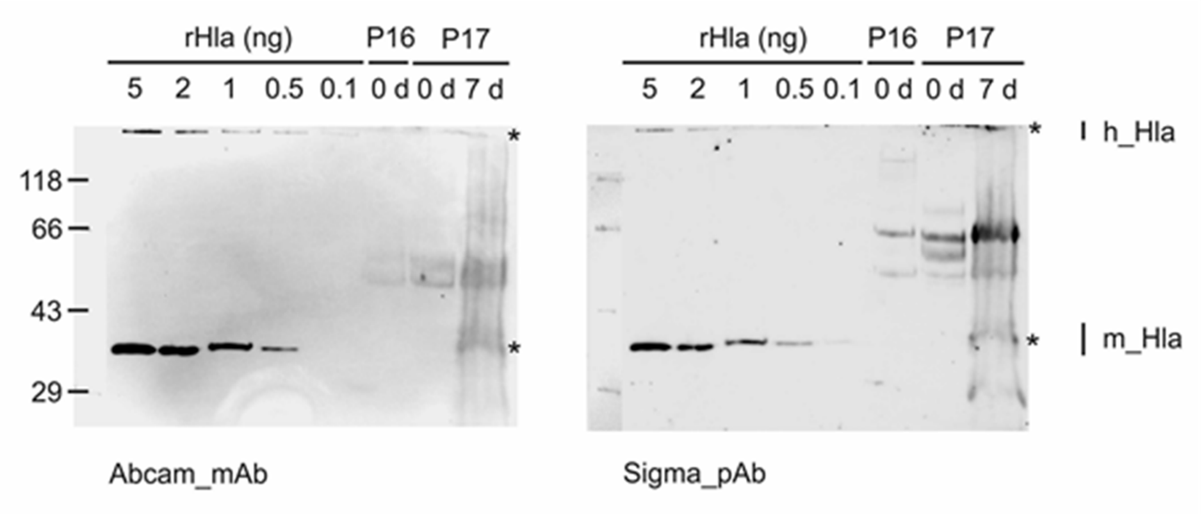

Figure S1. Detection and semi-quantification of monomeric Hla in deep tracheal aspirate (DTA) samples from patients with sepsis by semi-quantitative Western blotting. Shown is an example blot that had been incubated with the anti-Hla monoclonal antibody from Abcam (image on the left) and, upon stripping, with the anti-Hla polyclonal antibody from Sigma (image on the right). Five lanes of this blot were loaded with different amounts (0.1 to 5 ng) of recombinant Hla to compare their signal intensities with those of the DTA samples for semi-quantification of Hla content of each sample. The patient numbers (P) correspond to those given in Supplemental Table 1. The stars point to positive signals: m_Hla – monomeric Hla; h_Hla – heptameric Hla.

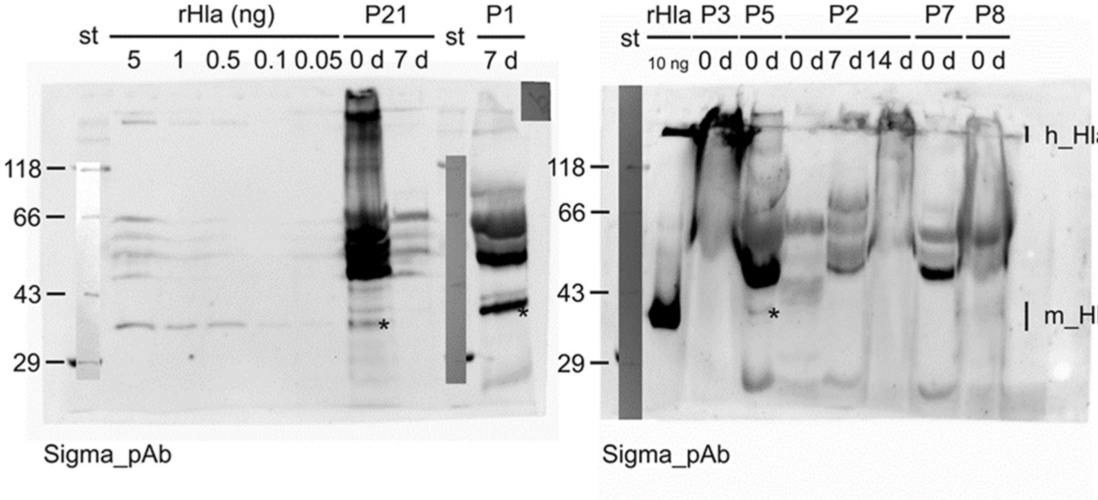

Figure S2. Detection and semi-quantification of monomeric Hla in deep tracheal aspirate (DTA) samples from patients with sepsis by semi-quantitative Western blotting. Shown are blots containing Hla-positive samples that had been incubated with the anti-Hla polyclonal antibody from Sigma. Five lanes of the blot on the left were loaded with different amounts (0.05 to 5 ng) of recombinant Hla to compare their signal intensities with those of the DTA samples for semi-quantification

of Hla content of each sample. The patient numbers (P) correspond to those given in Supplemental Table 1. The stars point to bands that correspond to monomeric Hla. m_Hla – monomeric Hla; h_Hla – heptameric Hla.

Table S1. List of deep tracheal aspirate (DTA) samples from hospitalized septic patients.

Patient #	Day of sampling	Putative origin of sepsis	Patient condition	Antibiotics at time of admission	Pathogen identified in blood culture	Causative Pathogens for sepsis	Monomeric Hla detected (this study)
1	7	Pneumonia	Septic shock	Meropenem	-	-	yes
2	0	Soft tissue infection	Severe sepsis	Meropenem, Anidulafungin	*Candida albicans*	-	no
	7						no
	14						no
3	0	unknown	Septic shock	Caspofungin, Vancomycin, Tigecycline, Metronidazole	-	*Enterococcus faecium*	no
4	0	Pneumonia	Severe sepsis	Ampicillin, Sulbactam	-	*Escherichia coli*	no
5	0	Intraabdominal infection	Septic shock	Meropenem	-	-	yes
6	0	Bone and soft tissue infection	Septic shock	Meropenem, Clindamycin, Cefuroxim	*Staphylococcus aureus*	-	no
	7						no
	14						no
7	0	Urogenital infection	Severe sepsis	Meropenem, Ceftriaxone	*Enterococcus faecalis, Proteus mirabilis*	-	no
8	0	Pleural empyema	Septic shock	Meropenem, Anidulafungin	-	Coagulase-negative *Staphylococcus*	no
9	7	Pneumonia	Septic shock	Linezolid	*Staphylococcus aureus* (MRSA)	-	no
10	0	Pneumonia	Severe sepsis	Piperacillin, Tazobactam	-	*Escherichia coli, Candida albicans*	no
	7						no
	14						no
11	0	Bone and soft tissue infection	Septic shock	Linezolid, Rifampicin	-	*Staphylococcus aureus* (MRSA)	no
	7						no
	14						no
12	0	Intraabdominal infection	Septic shock	Meropenem	-	*Pseudomonas, Enterococcus faecalis*	no
	7						no
	14						no
13	0	CNS infection	Severe sepsis	Cefotaxime, Flucloxacillin, Metronidazole	*Streptococcus pneumoniae*	-	no
	7						no
14	0	Pneumonia	Septic shock	Meropenem	-	*Escherichia coli*	no
15	0	Pneumonia	Septic shock	Meropenem, Linezolid,	-	*Klebsiella pneumoniae,*	no

				Clarithromycin		*Proteus mirabilis, Enterobacter cloacae compl.*	
16	0	Erysipelas	Severe sepsis	Meropenem, Penicillin	*Streptococcus pyogenes*	*Staphylococcus aureus*	yes
17	0	Prosthetic joint infection (PJI, knee)	Septic shock	Meropenem, Cefazolin, Linezolid	*Staphylococcus aureus*	-	no
	7						yes
	14						yes
18	0	Pneumonia	Severe sepsis	Piperacillin, Tazobactam, Clarithromycin	-	-	no
19	0	Intraabdominal infection	Septic shock	Meropenem, Linezolid	*Staphylococcus aureus* (MRSA)	-	no
20	0	Pneumonia	Septic shock	Meropenem	*Klebsiella pneumoniae*	-	no
21	0	Endocarditis	Septic shock	Meropenem	*Staphylococcus aureus*	-	yes
	7						no
22	0	Septic bursitis	Septic shock	Meropenem, Vancomycin	*Staphylococcus aureus*	-	no
